# Indications and Outcomes of Laparoscopic Versus Robotic Conversional Bariatric Surgery: An MBSAQIP Study

**DOI:** 10.1007/s11695-025-07886-6

**Published:** 2025-05-07

**Authors:** Juan S. Barajas-Gamboa, Mohammed Sakib Ihsan Khan, Kevin Zhan, Thomas H. Shin, Valentin Mocanu, Gustavo Romero-Velez, Andrew T. Strong, Salvador Navarrete, Carlos Abril, Juan Pablo Pantoja, A. Daniel Guerron, John Rodriguez, Ricard Corcelles, Matthew Kroh, Jerry T. Dang

**Affiliations:** 1 Cleveland Clinic Abu Dhabi, Abu Dhabi, United Arab Emirates; 2 Cleveland Clinic, Cleveland, United States; 3 College of Medicine and Health Sciences, Khalifa University, Abu Dhabi, United Arab Emirates; 4 Cumming School of Medicine, University of Calgary, Alberta, Canada; 5 Department of Surgery, University of Virginia, Charlottesville, VA USA

**Keywords:** Conversional bariatric surgery, Robotic surgery, Laparoscopic techniques, MBSAQIP, Surgical outcomes

## Abstract

**Background:**

Conversional bariatric surgeries (CBS) are performed using laparoscopic and robotic techniques, but comprehensive data comparing these approaches remains scarce.

**Objective:**

To compare the indications and outcomes of laparoscopic versus robotic CBS.

**Methods:**

The MBSAQIP database was retrospectively analyzed from 2020 to 2022, comparing laparoscopic and robotic CBS. Primary outcomes were 30-day serious complications and mortality.

**Results:**

Of 72,189 CBS procedures, 75.4% were laparoscopic and 24.6% robotic. Mean age and BMI were similar between groups. The most common indications for both approaches were reflux, weight regain, and inadequate weight loss, with reflux being more prevalent in robotic CBS (38.3% vs 33.2%). Sleeve-to-bypass was the most common procedure in both groups (35.8% laparoscopic, 44.2% robotic). Robotic CBS had longer mean operative times (165.4 vs 121.7 min, *p* < 0.001) and slightly longer hospital stays (1.7 vs 1.6 days, *p* < 0.001). The rate of serious complications was slightly higher for robotic CBS, though not statistically significant (6.5% vs 6.1%, *p* = 0.08). Robotic CBS had higher rates of leak (0.9% vs 0.7%, *p* = 0.071), reoperation (2.8% vs 2.6%, *p* = 0.138), and readmission (6.7% vs 5.4%, *p* < 0.001). Mortality rates were similar (0.1% for both, *p* = 0.942).

**Conclusions:**

Both laparoscopic and robotic CBS show similar safety profiles with comparable mortality rates. However, robotic CBS was associated with longer operative times, slightly longer hospital stays, and higher readmission rates. These findings suggest that the choice between approaches should consider individual patient factors and institutional expertise.

## Introduction

Obesity is an escalating global pandemic affecting millions worldwide [1]. Bariatric surgery has emerged as the most effective method for treating obesity [1–6]. Among the various bariatric procedures, Sleeve Gastrectomy (SG) and laparoscopic Roux-en-Y gastric bypass (RYGB) are the most common, accounting for 60.4% and 29.5% of all procedures, respectively^2^, [7–9]. While RYGB is considered the most established procedure for weight loss surgeries, SG is often preferred due to its lower complication rates, shorter operative time, and reduced risk of micronutrient deficiencies [6, 10]. However, SG is more likely to result in gastroesophageal reflux disease (GERD), recurrent weight gain, and suboptimal initial response compared to RYGB, potentially leading to the need for further conversional surgeries [6, 11].

Conversional bariatric surgery (CBS) rates have been observed to be as high as 12.2% after a 10-year period [1]. The most frequent revisional surgeries for these complications include RYGB, single-anastomosis duodeno-ileal bypass (SADI), and biliopancreatic diversion with duodenal switch (BPD-DS) [1]. A systematic review by Lee et al. concluded that conversions from SG to RYGB, SADI, and BPD-DS are comparably safe [12]. In recent years, the application of robotic platforms in bariatric surgery has gained popularity, offering several benefits, especially in complex surgeries. These advantages include enhanced ergonomics, three-dimensional stereotactic vision, and seven degrees of flexibility in the instruments. This flexibility provides surgeons with the ability to perform precise dissections in narrow spaces and execute accurate intracorporeal suturing [13, 14].

Despite the increasing adoption of robotic platforms for both primary and CBS, there is a scarcity of studies analyzing the outcomes and indications of laparoscopic and robotic CBS using prospective national multi-center databases [14–16]. Most existing evidence is limited to single-center studies and small series [14]. Clapp et al. and Nasser et al. utilized the extensive dataset from the Metabolic and Bariatric Surgery Accreditation and Quality Improvement Program (MBSAQIP) spanning 2015 to 2017 to compare laparoscopic and robotic CBS [15, 16]. However, the MBSAQIP data from 2020 added some additional details which were not available previously. Seton et al. reported their comparisons using MBSAQIP data from 2020 alone, despite the availability of data extending up to 2022 [17].

Therefore, the objective of this study is to assess the indications and outcomes of laparoscopic and robotic CBS, employing data from MBSAQIP from 2020 to 2022. By analyzing this comprehensive dataset, we aim to provide valuable insights into the comparative efficacy and safety of these two surgical approaches in conversional bariatric procedures.

## Methods

### Study Design and Population

This study is a retrospective study using prospectively collected data. This study included laparoscopic and robotic procedures from patients who underwent conversional bariatric surgery. The main objectives of this study were to assess the 30-day rates of serious complications and mortality. Serious complications were defined as a composite variable, indicating a patient experiencing one or more of the following within 30 days post-surgery: anastomotic leak, postoperative bleeding, reoperation, non-operative intervention, cardiac arrest, myocardial infarction (MI), cardiopulmonary resuscitation, pneumonia, unplanned intubation, acute kidney injury, venous thromboembolism (VTE), deep surgical site infection, sepsis, or cerebrovascular accident.

It is important to note that primary RYGB procedures that underwent subsequent surgery were not included in this analysis. This is due to the MBSAQIP database's classification system, which typically categorizes alterations to RYGB as'revisions'rather than'conversions.'This distinction in terminology within the database resulted in the exclusion of RYGB as a primary surgery in our conversion cohort, which should be considered when interpreting our results. It should be noted that our analysis includes a small proportion of cases with indications that may represent emergency situations (gastrointestinal tract bleeding, fistula, perforation, stricture/obstruction, and ulceration without perforation), collectively comprising 1.31% of the total cohort. These were maintained in the analysis to reflect the complete spectrum of conversion indications encountered in clinical practice.

### Ethics Approvals

This study was reviewed and classified as exempt by the Institutional Review Board (IRB) because the data were anonymous.

### Data Source

The Metabolic and Bariatric Surgery Accreditation and Quality Improvement Program (MBSAQIP) database registry was reviewed and analyzed. Only data from 2020 to 2022 were included, as a 2020 modification added details on revisional surgery that were not previously reported. While earlier MBSAQIP data (2015–2019) was available, we specifically focused on the 2020–2022 period due to more consistent and complete coding of robotic approaches during this timeframe, which reduced the risk of surgical approach misclassification. This decision prioritized data quality and specificity over extended timeframe, though we acknowledge the statistical power advantages that could have been gained from a longer study period.

### Patient Variables

Basic demographic data such as age, sex, race, and body mass index (BMI) were collected. Patient comorbidities included diabetes, hypertension, gastroesophageal reflux disease (GERD), chronic obstructive pulmonary disease (COPD), hyperlipidemia, chronic steroid use, chronic kidney disease, dialysis dependency, venous stasis, preoperative therapeutic anticoagulant use, and obstructive sleep apnea (OSA). Patient history covered previous venous thromboembolism (VTE), myocardial infarction (MI), percutaneous coronary intervention, and major cardiac surgery. Functional status variables included preoperative functional status (classified as independent, partially dependent, or fully dependent) and the American Society of Anesthesiologists (ASA) Physical Status classification.

### Statistical Analysis

The descriptive categorical data were expressed as percentages, while continuous data were presented as mean and standard deviation (SD). Non-parametric continuous data were expressed using median and interquartile range (IQR). Univariate analyses were conducted to identify baseline differences between groups, utilizing chi-squared tests for categorical data and independent sample t-tests for continuous data. Univariate logistic regression analysis was performed to identify predictive factors patients undergoing a robotic compared to laparoscopic CBS approach. The available case method addressed missing data, as all variables had less than 5% missingness. Patient factors and operative time were included in the model. Any variable with a *p*-value < 0.05 in univariate analysis was included in the logistic regression analysis. Variables were checked for multicollinearity using the variable inflation factor. All statistical analyses were conducted using STATA 17 statistical software (StataCorp, College Station, TX, USA).

## Results

### Patient Demographics

A total of 72,189 patients underwent conversional bariatric surgery, with 54,428 (75.4%) undergoing laparoscopic CBS and 17,761 (24.6%) undergoing robotic CBS. The mean age at conversion was slightly higher for laparoscopic CBS (48.5 ± 10.7 years) compared to robotic CBS (48.2 ± 10.7 years, p = 0.001). The majority of patients in both groups were female (87.8% in laparoscopic, 88.4% in robotic, *p* = 0.049) and white (65.3% in laparoscopic, 63.9% in robotic, *p* < 0.001). The mean body mass index was significantly higher in the robotic CBS group (41.5 ± 8.6 kg/m2) compared to the laparoscopic group (41.0 ± 8.4 kg/m2, *p* < 0.0001), though this difference, while statistically significant, is likely not clinically meaningful. Most patients in both groups had an independent functional status (99.4% laparoscopic, 99.3% robotic, *p* = 0.670) and were classified as ASA class 2 or 3 (97.2% laparoscopic, 96.9% robotic, *p* < 0.001).

Regarding comorbidities, the laparoscopic CBS group had a higher prevalence of smokers (5.2% vs 4.7%, *p* = 0.012) and hypertension (12.3% vs 8.4%, *p* = 0.024), while the robotic CBS group had higher rates of diabetes (15.3% vs 14.3%, *p* = 0.002), gastroesophageal reflux disease (58.8% vs 52.9%, *p* < 0.001), chronic obstructive pulmonary disease (1.3% vs 1.1%, *p* = 0.015), hyperlipidemia (21% vs 19.45%, *p* < 0.001), and obstructive sleep apnea (27.3% vs 25.6%, *p* < 0.001) (Table 1).

**Table 1:** Patient characteristics

	Laparoscopic CBS *n* = 54,428	Robotic CBS *n* = 17,761	*p-value*
Age, years mean ± SD	48.5 ± 10.7	48.2 ± 10.7	0.001
Female	47,801 (87.8)	15,697 (88.4)	0.049
Race White Black Other	35,553 (65.3) 12,441 (22.9) 6,434 (11.8)	11,347 (63.9) 4,306 (24.2) 2,108 (11.9)	< 0.001
Body mass index (kg/m [2]) mean ± SD	41.0 ± 8.4	41.5 ± 8.6	< 0.0001
Functional Status Independent Partially dependent Totally dependent	54,083 (99.4) 274 (0.5) 17 (0.03)	17,637 (99.3) 99 (0.6) 5 (0.03)	0.670
American Society of Anesthesiologists class 1 2 3	13,671 (25.1) 39,220 (72.1) 1404 (2.6)	4,016 (22.6) 13,191 (74.3) 547 (3.1)	< 0.001
Smoker	2,831 (5.2)	839 (4.7)	0.012
Diabetes None or diet-controlled Non-insulin-dependent Insulin-dependent	46,666 (85.7) 1,986 (3.7) 5,776 (10.6)	15,039 (84.7) 698 (3.9) 2,024 (11.4)	0.002
Hypertension	6,707 (12.3)	1,492 (8.4)	0.024
Gastroesophageal reflux disease	28,810 (52.9)	10,439 (58.8)	< 0.001
Chronic obstructive pulmonary disease	576 (1.1)	227 (1.3)	0.015
Hyperlipidemia	10,585 (19.45)	3,729 (21)	< 0.001
Chronic steroids	434 (0.8)	94 (0.5)	0.835
Renal insufficiency	214 (0.4)	82 (0.5)	0.215
Dialysis dependent	95 (0.2)	29 (0.2)	0.753
History of PE	1093 (2.0)	240 (1.91)	0.436
History of venous thromboembolism	1,996 (3.7)	606 (3.4)	0.113
Venous stasis	321 (0.6)	101 (0.6)	0.749
Preoperative therapeutic anticoagulant use	1,833 (3.4)	642 (3.6)	0.116
Obstructive sleep apnea	13,952 (25.6)	4,857 (27.3)	< 0.001
History of myocardial infarction	560 (1)	193 (1.1)	0.511
Previous percutaneous coronary intervention	733 (1.3)	280 (1.6)	0.024
Previous major cardiac surgery	505 (0.9)	143 (0.8)	0.132
IVC filter	219 (0.40)	75 (0.42)	0.718

*CBS* Conversional Bariatric Surgery, *SD* Standard Deviation, *PE* Pulmonary Embolism, *IVC* Inferior Vena Cava

### Most Common Primary Procedures Undergoing Conversion

Among the 51,138 primary procedures undergoing conversion, sleeve gastrectomy (SG) conversion procedures were the most common, totaling 30,664 cases. Of these, the majority (89.3%) were SG to Roux-en-Y gastric bypass (RYGB) conversions, with 19,533 (90.3%) performed laparoscopically and 7,855 (86.0%) robotically. SG to biliopancreatic diversion (BPD) conversions accounted for 6.2% of sleeve conversion procedures, while SG to single anastomosis duodenal-ileal bypass (SADI) conversions represented 4.4%. Band procedures were the second most common primary procedures undergoing conversion, totaling 19,758 cases. Among these, band to SG was the most frequent (59.4%), with 9,098 (61.0%) performed laparoscopically and 2,638 (54.4%) robotically. Band to RYGB conversions accounted for 37.6% of band conversion procedures. Vertical banding gastroplasty (VBG) conversion procedures were less common, with only 716 cases. The vast majority of these (92.1%) were VBG to RYGB conversions. Overall, laparoscopic approaches were more common across all types of CBS procedures, accounting for 72.4% of the total, compared to 27.5% for robotic approaches (Table 2).

**Table 2:** Most common primary procedures undergoing conversion

	**Laparoscopic CBS**	**Robotic CBS**	**Total Procedures**
**Sleeve Conversion Procedures (*n*,%)**			30,664
SG to RYGB	19,533 (90.3)	7,855 (86.0)	27,388 (89.3)
SG to SADI	868 (4.1)	480 (5.3)	1,348 (4.4)
SG to BPD	1,228 (5.6)	700 (7.7)	1,928 (6.2)
** Subtotal**	**21,629 (100.0)**	**9,035 (100.0)**	**30,664 (100.0)**
**Band Conversion Procedures (*n*,%)**			19,758
Band to SG	9,098 (61.1)	2,638 (54.4)	11,736 (59.6)
Band to RYGB	5,396 (36.1)	2,046 (42.1)	7,442 (37.6)
Band to SADI	107 (0.7)	50 (1.2)	157 (0.7)
Band to BPD	308 (2.1)	115 (2.3)	423 (2.1)
** Subtotal**	**14,909 (100.0)**	**4,849 (100.0)**	**19,758 (100.0)**
**VBG Conversion Procedures** **(*n*,%)**			716
VBG to RYGB	465 (91.5)	195 (93.7)	660 (92.2)
VBG to SG	43 (8.5)	13 (6.3)	56 (7.8)
** Subtotal**	**508 (100.0)**	**208 (100.0)**	**716 (100.0)**
** Total**	**37,046 (100.0)**	**14,092 (100.0)**	**51,138 (100.0)**

*CBS* Conversional Bariatric Surgery, *SG* Sleeve Gastrectomy, *RYGB* Roux-en-Y Gastric Bypass, *SADI* Single Anastomosis Duodenal-Ileal Bypass, *BPD* Biliopancreatic Diversion, *Band* Adjustable Gastric Banding, *VBG* Vertical Banded Gastroplasty

### Conversion Indications and Intraoperative Findings

The most common indication for CBS in both laparoscopic and robotic approaches was gastroesophageal reflux disease (GERD), with a significantly higher prevalence in robotic CBS (38.3% vs 33.3%, *p* < 0.0001). This was followed by recurrent weight gain (31.9% laparoscopic, 32.0% robotic, *p* = 0.7022) and suboptimal initial response (20.9% laparoscopic, 18.7% robotic, *p* < 0.0001). Laparoscopic CBS had significantly higher rates of indications such as abdominal pain (0.4% vs 0.3%, *p* = 0.018), adhesions (1.1% vs 0.2%, *p* < 0.0001), dysphagia (2.9% vs 2.4%, *p* = 0.0049), and patient intolerance (1.0% vs 0.7%, *p* < 0.0001). Conversely, robotic CBS had a significantly higher rate of anastomotic or staple line leak as an indication (0.2% vs 0.1%, *p* < 0.0001). Regarding intraoperative findings, robotic CBS was associated with significantly longer mean operative times compared to laparoscopic CBS (165.5 ± 74.2 min vs 121.8 ± 70.04 min, *p* < 0.001). The mean length of hospital stay was also slightly longer for robotic CBS (1.73 ± 2.36 days) compared to laparoscopic CBS (1.60 ± 2.11 days, *p* < 0.001) (Table 3).

**Table 3:** Indications for surgery and intraoperative findings

	Laparoscopic CBS *n* = 54,428	Robotic CBS *n* = 17,761	*p-value*
Indication for surgery			
Abdominal pain	157 (0.4)	39 (0.3)	0.018
Adhesions	461 (1.1)	32 (0.2)	< 0.0001
Anastomotic or staple line leak	33 (0.1)	37 (0.2)	< 0.0001
Dumping	10 (0.02)	8 (0.1)	0.1058
Dysphagia	1155 (2.9)	370 (2.4)	0.0049
Fluid, electrolyte, or nutritional depletion	38 (0.1)	14 (0.1)	0.9340
Gastroesophageal reflux disease (GERD)	13,480 (33.3)	5,875 (38.3)	< 0.0001
Gastrointestinal tract bleeding	2 (0.005)	2 (0.01)	0.3118
Gastrointestinal tract fistula	37 (0.1)	12 (0.1)	0.6439
Gastrointestinal tract perforation	18 (0.04)	5 (0.03)	0.5403
Gastrointestinal tract stricture or obstruction	447 (1.1)	164 (1.1)	0.7407
Gastrointestinal tract ulcer without perforation	36 (0.1)	11 (0.1)	0.5355
Hypoglycemia	12 (0.03)	8 (0.1)	0.2078
Suboptimal initial response	8,470 (20.9)	2,871 (18.7)	< 0.0001
Mechanical malfunction	877 (2.2)	308 (2)	0.2602
Nausea and/or vomiting	438 (1.1)	130 (0.8)	0.0146
Other (not listed)	1,059 (2.6)	302 (2)	< 0.0001
Patient intolerance	412 (1)	99 (0.7)	< 0.0001
Patient non-compliance	14 (0.03)	2 (0.01)	0.1805
Persistent comorbidities	440 (1.1)	130 (0.9)	0.0128
Recurrent weight gain	12,926 (31.9)	4,913 (32)	0.7022
Operative time minutes ± SD	121.8 ± 70.04	165.5 ± 74.2	< 0.001
Length of stay days ± (SD)	1.60 (2.11)	1.73 (2.36)	< 0.001

*CBS* Conversional Bariatric Surgery, *GERD* Gastroesophageal Reflux Disease, *SD* Standard Deviation

We conducted additional analyses to characterize the primary procedures associated with specific conversion indications. For dumping syndrome (*n* = 18), Roux-en-Y gastric bypass was the most common primary procedure (55.6%), followed by adjustable gastric banding and biliopancreatic diversion with duodenal switch (16.7% each). For dysphagia (*n* = 1,525), adjustable gastric banding was predominant (66.9%), followed by sleeve gastrectomy (27.7%). For fluid, electrolyte, or nutritional depletion (*n* = 52), sleeve gastrectomy was most common (48.1%), followed by biliopancreatic diversion with duodenal switch (17.3%). For hypoglycemia (*n* = 20), sleeve gastrectomy and Roux-en-Y gastric bypass were equally represented (35% each). Mechanical malfunction (*n* = 1,185) referred to device-related complications and was predominantly associated with adjustable gastric banding (91.9%). Patient intolerance (*n* = 511), defined as persistent food intolerance including continued vomiting without obstruction and maladaptive eating patterns, was primarily associated with adjustable gastric banding (89.8%). Patient non-compliance (*n* = 16), referring to documented inability to adhere to required dietary and behavioral modifications, was most commonly following adjustable gastric banding (56.3%). For the'other'category (*n* = 1,361), the primary procedures were adjustable gastric banding (49.5%) and sleeve gastrectomy (38.4%).

### Postoperative Complications and Mortality

The overall rate of serious complications was slightly higher in robotic CBS compared to laparoscopic CBS, although the difference was not statistically significant (6.5% vs 6.2%, *p* = 0.082). Robotic CBS showed no significant differences in anastomotic leak rates (0.9% vs 0.8%, *p* = 0.071) or reoperation rates (2.8% vs 2.6%, *p* = 0.138), but was associated with significantly higher readmission rates compared to the standard approach (6.8% vs 5.5%, *p* < 0.001). Notably, robotic CBS showed significantly higher rates of non-operative interventions (2.2% vs 1.9%, *p* = 0.03), venous thromboembolism (0.5% vs 0.4%, *p* = 0.005), and sepsis (0.4% vs 0.3%, *p* = 0.015). Conversely, laparoscopic CBS had a higher rate of postoperative bleeding (1.7% vs 1.4%, *p* = 0.024). Other complications, including cardiac events, pneumonia, acute kidney injury, deep surgical site infections, wound disruptions, unplanned intubations, and cerebrovascular accidents, showed no statistically significant differences between the two approaches. The mortality rates were similar between robotic and laparoscopic CBS (0.1% for both, *p* = 0.942), indicating comparable safety profiles in terms of patient survival (Table 4).

**Table 4:** Postoperative complications

	Laparoscopic CBS *n* = 54,428	Robotic CBS *n* = 15,332	*p-value*
Leak	429 (0.8)	165 (0.9)	0.071
Bleeding	909 (1.7)	253 (1.4)	0.024
Reoperation	1417 (2.6)	499 (2.8)	0.138
Non-operative intervention	1038 (1.9)	385 (2.2)	0.03
Readmission	2990 (5.5)	1199 (6.8)	< 0.001
Cardiac events	66 (0.1)	29 (0.2)	0.180
Pneumonia	230 (0.4)	91 (0.5)	0.118
Acute kidney injury	68 (0.1)	29 (0.2)	0.226
Venous thromboembolism	198 (0.4)	92 (0.5)	0.005
Deep surgical site infection	680 (1.2)	249 (1.4)	0.117
Wound disruption	54 (0.1)	18 (0.1)	0.847
Sepsis	169 (0.3)	77 (0.4)	0.015
Unplanned intubations	106 (0.19)	34 (0.19)	0.238
Cerebrovascular accident	13 (0.02)	5 (0.03)	0.754
Serious complications	3354 (6.2)	1159 (6.5)	0.082
Death	81 (0.1)	26 (0.1)	0.942

*CBS* Conversional Bariatric Surgery

When examining trends across the study period (2020–2022), we observed a notable increase in the proportion of robotic CBS procedures over time (17.5% in 2020, 23.2% in 2021, and 31.4% in 2022). For both approaches, serious complications demonstrated a favorable trend: laparoscopic CBS serious complications decreased from 6.39% in 2020 to 5.64% in 2022 (*p* = 0.002), while robotic CBS serious complications decreased from 7.64% in 2020 to 6.14% in 2022 (*p* = 0.009). Similarly, length of stay decreased over the study period for both laparoscopic (from 1.69 days in 2020 to 1.53 days in 2022) and robotic approaches (from 1.84 days in 2020 to 1.68 days in 2022).

### Logistic Regression for Robotic Surgical Approach

Univariate logistic regression analysis was performed to examine associations between various factors and the use of robotic versus laparoscopic approach for the three most common CBS procedures: SG to RYGB, band to SG, and band to RYGB.

Preoperative Factors: Age was not significantly associated with the surgical approach in any of the procedures. Body mass index (BMI) showed a significant, albeit small, positive association with robotic approach only for band to SG conversions (OR = 1.001, 95% CI: 1.0010–1.0120, *p* = 0.020). Higher preoperative albumin levels were significantly associated with lower odds of undergoing a robotic approach across all three procedures, indicating patients with lower albumin levels were more likely to undergo robotic conversions: SG to RYGB (OR = 0.834, 95% CI: 0.7747–0.8981, *p* < 0.001), band to SG (OR = 0.766, 95% CI: 0.6770–0.8672, *p* < 0.001), and band to RYGB (OR = 0.822, 95% CI: 0.7093–0.9537, *p* = 0.010) (Table 5).

**Table 5:** Univariate logistic regression for features associated with patients undergoing a robotic compared to laparoscopic CBS approach

Risk Factor	SG to RYGB Odds ratio [95% CI]	SG to RYGB pvalue	SG to RYGB standard error	Band to SG Odds ratio [95% CI]	Band to SG pvalue	Band to SG standard error	Band to RYGB Odds ratio [95% CI]	Band to RYGB pvalue	Band to RYGB standard error
Age	1.001 [0.9985–1.0035]	0.421	0.0012766	1.001 [0.9972–1.0056]	0.520	0.0021658	1.005 [1.0000–1.0102]	0.053	0.0026131
BMI	0.999 [0.9952–1.0019]	0.387	0.0017175	1.001 [1.0010–1.0120]	0.020	04.0027955	0.996 [0.9892–1.0028]	0.245	0.0034693
Albumin	0.834 [0.7747- 0.8981]	< 0.001	0.03146	0.766 [0.6770–0.8672]	< 0.001	0.0004408	0.822 [0.7093–0.9537]	0.010	0.0621096
Operative time	1.001 [1.0063- 1.0071]	< 0.001	0.0002055	1.010 [1.0092–1.0109]	< 0.001	0.0004408	1.001 [1.0068–1.0082]	< 0.001	0.0003615
Length of stay	0.985 [0.9702- 1.001]	0.060	0.007775	1.047 [1.0156–1.0791]	0.003	0.0161923	0.984 [0.9528–1.0165]	0.332	.0162492

*CBS* Conversional Bariatric Surgery, *SG* Sleeve Gastrectomy, *RYGB* Roux-en-Y Gastric Bypass, *CI* Confidence Interval, *BMI* Body Mass Index

Intraoperative and Postoperative Outcomes: Robotic approach was associated with significantly longer operative times for all procedures: SG to RYGB (OR = 1.001, 95% CI: 1.0063–1.0071, *p* < 0.001), band to SG (OR = 1.010, 95% CI: 1.0092–1.0109, *p* < 0.001), and band to RYGB (OR = 1.001, 95% CI: 1.0068–1.0082, *p* < 0.001). Length of hospital stay was significantly associated with robotic approach only for band to SG conversions (OR = 1.047, 95% CI: 1.0156–1.0791, *p* = 0.003) (Table 5).

## Discussion

Our study, utilizing the MBSAQIP data from 2020 to 2022, reveals several key findings regarding laparoscopic and robotic CBS. We observed a higher prevalence of laparoscopic CBS (75.4%) compared to robotic CBS (24.6%). GERD emerged as the primary indication for both approaches, with a significantly higher prevalence in robotic CBS (38.3% vs 33.3%, *p* < 0.0001), followed by recurrent weight gain (31.9% laparoscopic, 32.0% robotic) and suboptimal initial response (20.9% laparoscopic, 18.7% robotic). Interestingly, our analysis revealed some differences in the indications for laparoscopic versus robotic CBS. Laparoscopic CBS had significantly higher rates of indications such as abdominal pain (0.4% vs 0.3%, *p* = 0.018), adhesions (1.1% vs 0.2%, *p* < 0.0001), dysphagia (2.9% vs 2.4%, *p* = 0.0049), and patient intolerance (1.0% vs 0.7%, *p* < 0.0001), while robotic CBS had a significantly higher rate of anastomotic or staple line leak as an indication (0.2% vs 0.1%, *p* < 0.0001).

Notably, robotic CBS was associated with significantly longer mean operative times (165.5 vs 121.8 min, *p* < 0.001) and slightly longer hospital stays (1.73 vs 1.60 days, *p* < 0.001) compared to laparoscopic CBS. While robotic CBS showed no significant differences in anastomotic leak rates (0.9% vs 0.8%, *p* = 0.071) or reoperation rates (2.8% vs 2.6%, *p* = 0.138), it was associated with significantly higher readmission rates compared to the standard approach (6.8% vs 5.5%, *p* < 0.001). Robotic CBS also showed significantly higher rates of non-operative interventions (2.2% vs 1.9%, *p* = 0.03), venous thromboembolism (0.5% vs 0.4%, *p* = 0.005), and sepsis (0.4% vs 0.3%, *p* = 0.015). These findings suggest that while robotic CBS may offer advantages in certain scenarios, it is also associated with some increased risks that need to be carefully considered in patient selection and perioperative management.

It is important to note that selection bias likely influenced our results, as patients who underwent robotic conversional procedures had a slightly higher BMI (41.53 vs. 41.04, *p* < 0.0001)—a difference that is statistically but not clinically significant—and higher rates of several comorbidities including GERD (58.8% vs. 52.9%, *p* < 0.001), COPD (1.28% vs. 1.06%, *p* = 0.015), and hyperlipidemia (21.0% vs. 19.5%, *p* < 0.001). These findings suggest that surgeons may be selecting the robotic approach for patients with more complex conditions or challenging anatomical characteristics, which could impact surgical outcomes independent of the technical approach. The observed differences in complication rates between robotic and laparoscopic approaches should therefore be interpreted with caution, as they may be confounded by these baseline differences in patient characteristics rather than representing a direct effect of the surgical approach itself. A comprehensive multivariate analysis accounting for all relevant comorbidities would be necessary to determine the independent effect of surgical approach on outcomes, representing an important direction for future research. This selection bias should be considered when interpreting the differences in outcomes between robotic and laparoscopic conversional procedures. Additionally, the consistent association between lower preoperative albumin levels and higher likelihood of robotic approach further suggests selection of more nutritionally compromised patients for robotic procedures, possibly due to anticipated technical challenges.

The apparent contradiction of higher leak rates in the robotic group despite lower smoking rates may be explained by the higher overall comorbidity burden in these patients. Furthermore, the consistently longer operative times observed with robotic procedures (165.5 vs 121.8 min, *p* < 0.001) may reflect multiple factors related to surgeon experience. These could include experienced laparoscopic surgeons still navigating the learning curve with robotic technology, or possibly earlier adoption of robotic approaches by surgeons at earlier stages in their career trajectories. The MBSAQIP database does not include information on surgeon experience, case volume, or where surgeons are on their individual learning curves with robotic technology, limiting our ability to directly evaluate these factors. This represents an important area for future research.

Our analysis of yearly trends from 2020 to 2022 reveals interesting patterns that may reflect learning curve effects. The relative decrease in serious complications was more pronounced in the robotic group (19.6% reduction) compared to the laparoscopic group (11.7% reduction). This larger improvement in the robotic cohort suggests that as institutions gained experience with robotic CBS, outcomes improved more substantially than with the more established laparoscopic approach. Despite these improvements, robotic CBS maintained higher complication rates and longer hospital stays throughout the study period, indicating that patient selection factors likely continue to play an important role in the observed outcome differences. The increasing proportion of cases performed robotically over the study period (from 17.5% in 2020 to 31.4% in 2022) further demonstrates the growing adoption of robotic techniques for these complex conversional procedures despite the longer operative times and potential for higher complication rates.

Our findings both align with and diverge from previous studies in this field. A large study on over 800,000 patients from the MBSAQIP dataset found longer operative times for robotic bariatric surgery, consistent with our results [18]. However, the literature on robotic bariatric surgery outcomes is mixed. While one study suggests higher rates of complications and readmissions for robotic-assisted bariatric surgery compared to laparoscopic surgery [19], a meta-analysis found no significant difference in post-operative complication rates between the two approaches except for higher reoperation rates in robotic RYGB patients [20]. Conversely, another review showed that robotics led to lower complications, lower leak rates, shorter operative times, and shorter learning curves for robotic bariatric surgery [21, 22].

Specific to revisional and conversional bariatric surgery, our findings partially align with previous studies. Clapp et al. reported longer operative times and hospital stays for robotic revisional bariatric surgery, but found no difference in serious complications [15]. Nasser et al. also reported longer operative times and identified several post-operative complications that were increased with robotic SG CBS and decreased complications in robotic RYGB CBS [16]. They also reported no difference in rates of mortality, readmission or reoperation between the two approaches [16]. However, a single cohort study by King et al. found robotic revisional bariatric surgery to have shorter lengths of hospital stay and non-significantly lower rates of postoperative complications [23].

Longer operation time and longer length of stay may be due to surgeons preferring robotic approaches for more complex patients and surgical procedures [24–26]. Longer operative times also are associated with higher rates of VTE post-operation [27] which was also significantly higher in the robotic CBS group for our study. Post-operative sepsis which was also increased in the robotic CBS group could also account for the increased hospital LOS [15]. The significantly longer operative times we observed with robotic CBS (165.5 vs 121.8 min, *p* < 0.001) likely have important cost implications, as operating room time is a major contributor to the overall cost of surgery. While the MBSAQIP database does not include financial data for direct cost analysis, the 36% increase in operative time combined with the higher capital and per-case costs associated with robotic technology suggests that robotic CBS may be considerably more expensive than laparoscopic approaches. Also, due to the popularity of robotic surgery, new procedures and technologies are constantly being developed [28]. As such, there is a learning curve of around 15–30 cases for robotic surgical skill to plateau [29, 30] which could result in an increase in initial operative time for surgeons as well as increased complications. This learning curve effect may be particularly relevant for conversional bariatric procedures, which are generally more complex than primary cases and may require more specialized training and experience to achieve optimal outcomes with robotic platforms [29, 30]. 

Our analysis included a small proportion of cases (1.31%) with indications suggesting potentially emergent conversions (gastrointestinal bleeding, fistula, perforation, stricture/obstruction, and ulceration). The inclusion of these cases provides a more comprehensive view of real-world practice, but this heterogeneity should be considered when interpreting our results, as emergency procedures inherently carry different risk profiles regardless of surgical approach. Given their small proportion in our cohort, these emergency indications are unlikely to significantly impact our overall findings.

Our study has several limitations. The MBSAQIP database lacks complication outcomes beyond 30 days post-surgery and does not include weight loss outcomes. Our decision to focus exclusively on 2020–2022 data, while improving data quality and specificity, limits our ability to observe longer-term trends in robotic adoption and outcomes. A comprehensive analysis including earlier years (2015–2019), while adjusting for the differences in data collection methodology, could provide additional insights in future studies. There may be selection bias, as more complex cases could be preferentially selected for either robotic or laparoscopic approaches. This selection bias makes it challenging to fully disentangle the independent effects of patient factors versus surgical approach on outcomes without more sophisticated statistical approaches. The database does not include healthcare cost data, which is relevant given the expenses associated with robotic systems in terms of equipment, maintenance, and training. Additionally, the dataset does not differentiate between experienced and novice surgeons, which could introduce variability in outcomes. Lastly, the specific model of robotic instrument used is not specified, which could influence patient outcomes as newer models become available.

Despite these limitations, our study's strengths lie in its large sample size, multi-institutional nature, and the use of recent data (2020–2022) that includes additional details on revisional surgery not previously available. Future research should focus on long-term outcomes beyond 30 days, including weight loss efficacy and resolution of comorbidities. Studies incorporating cost-effectiveness analyses would provide valuable insights for healthcare systems considering the adoption of robotic platforms. Additionally, prospective studies controlling for surgeon experience and specific robotic systems used could help clarify the impact of these factors on outcomes.

In conclusion, our study provides a comprehensive comparison of laparoscopic and robotic CBS using recent, large-scale data. While robotic CBS is associated with longer operative times and hospital stays, as well as higher rates of certain complications, it shows similar mortality rates to laparoscopic CBS. The choice between laparoscopic and robotic approaches should be based on individual patient factors, surgeon expertise, and institutional resources. As robotic technology continues to evolve, ongoing evaluation of its role in conversional bariatric surgery remains crucial.

## Data Availability

No datasets were generated or analysed during the current study.
